# Sharing responsibility: municipal health professionals’ approaches to goal setting with older patients with multi-morbidity – a grounded theory study

**DOI:** 10.1186/s12913-020-4983-3

**Published:** 2020-02-24

**Authors:** Jannike Dyb Oksavik, Ralf Kirchhoff, Maren Kristine Raknes Sogstad, Marit Solbjør

**Affiliations:** 1 Department of Health Sciences, Faculty of Medicine and Health Sciences, Norwegian University of Science and Technology, Ålesund, Norway; 2 The Centre for Care Research, and Department for Health Sciences, Faculty of Medicine and Health Sciences, Norwegian University of Science and Technology, Gjøvik, Norway; 3 Department of Public Health and Nursing, Faculty of Medicine and Health Sciences, Norwegian University of Science and Technology, Trondheim, Norway

**Keywords:** Health care delivery, integrated, Co-production, Collaborative goal setting, Health professionals, Multi-morbidity, Aged, 80 and over, Conceptual model

## Abstract

**Background:**

Recent health policy promoting integrated care emphasizes to increase patients’ health, experience of quality of care and reduce care utilization. Thus, health service delivery should be co-produced by health professionals and individual patients with multiple diseases and complex needs. Collaborative goal setting is a new procedure for older patients with multi-morbidity. The aim is to explore municipal health professionals’ experiences of collaborative goal setting with patients with multi-morbidity aged 80 and above.

**Methods:**

A qualitative study with a constructivist grounded theory approach. In total twenty-four health professionals from several health care services in four municipalities, participated in four focus group discussions.

**Results:**

Health professionals took four approaches to goal setting with older patients with multi-morbidity: motivating for goals, vicariously setting goals, negotiating goals, and specifying goals. When ‘motivating for goals’, they educated reluctant patients to set goals. Patients’ capacity or willingness to set goals could be reduced, due to old age, illness or less knowledge about the health system. Health professionals were ‘vicariously setting goals’ when patients did not express or take responsibility for goals due to adaptation processes to disease, or symptoms as cognitive impairment or exhaustion. By ‘Negotiating goals’, health professionals handled disagreements with patients, and often relatives, who expected to receive more services than usual care. They perceived some patients as passive or having unrealistic goals to improve health. ‘Specifying goals’ was a collaboration. Patients currently treated for one condition, set sub-goals to increase health. Patients with complex diseases prioritized one goal to maintain health. These approaches constitute a conceptual model of how health professionals, to varying extents, share responsibility for goal setting with patients.

**Conclusions:**

Goal setting for patients with multi-morbidity were carried out in an interplay between patients’ varying levels of engagement and health professionals’ attitudes regarding to what extents patients should be responsible for pursuing the integrated health services’ objectives. Even though goal setting seeks to involve patients in co-production of their health service delivery, the health services´ aims and context could restrict this co-production.

## Background

Recent health policy promoting integrated care emphasizes that health professionals and patients can co-create value when patients participate in formulating how their own health services should be delivered. Health professionals should, therefore, to a greater extent, collaborate with older patients when planning their care [1–3]. The number of persons over 80 years of age is increasing, and multi-morbidity, which is having two or more chronic diseases, is frequent in this group [4–7]. Healthcare for patients with multi-morbidity often comprises care from several services, from multidisciplinary health professionals, and from several clinical guidelines. Often, older patients experience functional decline and receive complex care over a long period of time. The goal for the care these patients receive is not always unified across care settings, where variation in goals between the health professionals involved, as well as between health professionals and patients, may occur [3–6, 8–10]. In order to resolve discrepancies between the opinions of patients and those of health professionals regarding health care delivery, it is recommended in national clinical guidelines for people with multi-morbidity that they should have the opportunity to collaborate with health professionals to formulate goals for own care [11, 12]. However, little is known about how health professionals initiate and practice collaborative goal setting with patients with multi-morbidity [13, 14].

Integrated care are structured efforts to provide coordinated, pro-active and multidisciplinary care, which is centred around individual patients’ preferences [1–3]. When health professionals add patients’ preferences in decisions about health service delivery, the services are co-produced [1]. Co-production at the individual level, is a collaborative process in which health professionals and patients share mutual information and define strategies for dealing with illness [15, 16]. A reciprocal contribution to co-production occurs when patients take greater responsibility for and actively collaborate in planning their own care, while health professionals involve and support patients to manage chronic conditions in daily life, based on the patients’ own values, preferences, and needs [1–3, 17, 18]. Through co-production, additional value is co-created [15, 16]. The primary value is improvement of the patient experience of service delivery. A secondary value is the reduction in the utilization of care services that can be achieved when health services help patients to live more independently [16, 19]. However, engaging older patients with multi-morbidity to actively collaborate with health professionals may be challenging due to their frail health, the changing severity of their diseases, and their complex care needs [2]. Previously, health professionals only to a limited extent have collaborated with patients on what matters to them [2, 20].

To overcome such difficulties, a specific form of collaborative goal setting has been suggested in order to attend to patients’ preferences and needs in the co-production of service delivery. Collaborative goal setting is ‘a process by which health professionals and patients agree on a health-related goal’ ([13], [20], p., 2). A health professional asks the patient to express needs and goals for care delivery according to the patient’s own definition of health. Goal setting can be carried out each time the patient receives a new health service in the care pathway [21]. The patient’s goals can be related to disease symptoms, physical functioning, or well-being; they can also be social goals or goals related to values of life [20].

Goal setting is a complex interactional activity in which health professionals play an important role [22]. A review indicates that health professionals perceive that goal setting increases collaboration with patients [8]. However, the process of negotiating and formulating specific goals is a challenging one, in which health professionals see a need to educate patients to succeed [23–25]. They perceive that the articulation of goals does not come naturally to all patients [23]. Health professionals are reserved about involving patients whom they perceive to be unmotivated or to be taking less responsibility for setting goals [8, 26], who have problems with communication or cognition [8], or who are perceived as less able to set goals [27]. Consequently, health professionals may perceive they should control goal setting by excluding specific patients or specific psychosocial goals in order to responsibly implement their professional knowledge as well as to respect time pressures and financial constraints [8, 26–29]. Palumbo [15, 19] suggests that co-destruction rather than co-creation of value can occur: the parties can be unaware of the clash of their interests or deliberately struggle to achieve benefits from the service provision. If the parties do not share common goals, their interactions do not co-create any additional value for patients in service delivery [15, 19].

There is limited research on goal setting for patients with multi-morbidity across settings [13, 14]. Previously, goal setting has been studied within neurological, rehabilitation, and in-patient settings [8, 9, 13, 26]. According to integrated care models, many Norwegian municipalities are implementing a procedure of goal setting in health care delivery for older patients [12]. Even though research exists on how health professionals carry out goal setting [23–28], few studies have conceptualized this for older patients in the municipal settings [13, 14], which have a health policy promoting co-production [1]. The aim of this study was to explore municipal health professionals’ experiences of interacting with patients with multi-morbidity aged 80 and above in collaborative goal setting.

## Methods

### Design

To explore health professionals’ experiences of interacting with older patients, a qualitative study using a constructivist grounded theory approach is particularly suitable [30]. Constructivist grounded theory focuses on actions and interactions, aiming for an abstracted understanding of experiences [30]. Constructivist grounded theory views the analysis as located in time, place, and situation [30], which is preferable when studying goal setting within a municipal context. We chose focus groups because we aimed to explore the experiences of a particular group in relation to a defined subject [31]. The interaction in focus groups can generate rich data by encouraging participants to explore and clarify individual and shared experiences and perspectives [31]. In constructivist grounded theory, the analysis begins after the first interview [30]. The application of this method allowed us to explore goal setting progressively by adapting subsequent focus group discussions in light of findings from earlier ones.

### Setting

#### The Norwegian context

In Norway, municipal integrated care for older people includes rehabilitation and long-term care, which takes place in community hospitals for rehabilitation, in nursing homes, or in patients’ homes. Patients can also receive reablement, which is a time-limited and intensive rehabilitation service delivered in patients’ homes. Reablement aims to improve patients’ physical abilities and maximize independence [32]. The amount and kind of services individual patients receive from municipal health services following a hospital stay is decided by an office for allocation of services (using a purchaser-provider model) or by municipal health service managers. Decisions are based on health professionals’ assessment of the patient’s functional level. Following the assignment of services to the patient, health professionals involve patients in discussions about how the services will be delivered [33]. Health professionals in municipalities who work with older patients often comprise nurses, auxiliary nurses, one physician, one physiotherapist, and an occupational therapist. These health professionals work in institutions or in patients’ homes. Patients aged 80 and over stay an average of 15 days in short-term or rehabilitation wards. They can receive four weeks of rehabilitation support in their homes [34]. Some patients transfer directly from the hospital to home, with or without home care services.

#### Study setting

The municipalities included in this study had implemented the goal setting procedure ‘What matters to you?’ [14] for 6–12 months prior to the focus group discussions. The procedure is a consultation in which a health professional identifies a patient’s goal for follow-up care in the municipality after hospital discharge [14, 35]. A goal is collaboratively set and documented for both the patient and the team of health professionals to work towards [21]. The procedure was implemented for all patients eligible for municipal health care. This study includes two rural (2000–3000 inhabitants) and two urban municipalities (with 40,000 and 70,000 inhabitants, respectively) in Western Norway. In each municipality, we included health professionals from several services: community hospital wards, rehabilitation wards, short-term wards in nursing homes, reablement teams, offices for allocation of services, and home care services.

### Recruitment and sample

The health professionals were purposively selected to represent a variety in occupations working in different clinical settings. Moreover, participants were eligible if having experiences with initiating goal setting by asking patients ‘What matters to you?’. They were recruited by a manager in each municipality, who invited health professionals to a focus group discussion in their workplace. In total, 27 participants were invited, but 3 did not attend due to illness. Each of the four groups consisted of 5–7 participants, for a total of 24 health professionals, including head nurses in nursing homes (4), head nurse in home care services (1), nurses (7), caseworker (1), auxiliary nurses (3), occupational therapists (2), physical therapists (4), physician (1), and one person without health education (1). Two of the focus groups had a male participant; the rest of the participants were females. Regarding their age, 5 of them were between 20 and 30 years old, 10 were between 30 and 40, 4 were between 40 and 50 and 5 were 50–65 years old. Their work experience within this area ranged from 6 months to 30 years.

### Data collection

We conducted four focus group discussions from September 2018 to February 2019. The focus groups took place without interruption in meeting rooms within participants’ workplaces. A semi-structured interview guide contained questions prompting health professionals to describe and discuss clinical situations they had experienced of goal setting for patients aged 80 and above after hospital stays. (Additional file – Interview guide). The discussions lasted approximately 90 min. They were audio recorded and later transcribed verbatim and anonymized by the first author. The first author wrote field notes and observations about the interactions in each group. We analyzed data after each interview. From the categories generated by our ongoing analysis, we derived theoretical sampling questions and added these to the interview guide for subsequent focus groups [30].

### Data analysis

Constructivist grounded theory explores processes as well as actions and interactions [30]. We used patient involvement as a sensitizing concept [30], which means that how health professionals experienced to involve patients, served as a point of departure for our analysis of health professionals’ interactions with patients. The concept patient involvement did not define or delimit how the data would be coded. In the initial coding, we divided the focus group data into small units and coded to explore health professionals’ actions [30]. Then, in focused coding, we merged initial codes that were similar and concentrated on frequent and significant codes. By the constant comparison method, we tested these codes against the rest of the data to develop the categories [30]. The categories related mainly to characteristics of patients, levels of collaboration, and how the municipal context influenced actions. The software NVivo version 12 supported focused coding. Table 1 shows an example from the coding.

**Table 1:** Coding from quotes to category

Quotation	Initial coding	Focused coding	Category
‘Some say, for example, that it is important for them to be able to walk and then we see that it is unlikely, we cannot take away their hope. Because then they may not want to be with us or lose all motivation. But we try in a way to orientate on something that is achievable while they are with us, for example to be able to go with aids or other things that may be important to them.’ (Occupational therapist, Group 1)			
	Perceiving the goal as too ambitious		
	Calculating consequences of addressing it		
	Reality-orientating	Reality-orientating	Negotiating goals
	Adjusting the goal downward		

Through theoretical coding, categories relating to one another and accounting for the data were included in a conceptual model [36] of approaches to goal setting. Memos were written throughout the process of analysis to guide and record the analysis [30]. Constructivist grounded theory recognizes the researcher as situated within the research process and acknowledges that several interpretations of the data are possible [30]. Thus, the authors, who come from different disciplinary backgrounds, discussed the interpretations regularly. We found that the fourth focus group validated the categories from the analysis of the first interviews. Due to this saturation in the categories [30], four focus groups were considered enough for development of the concept within this study.

## Results

Overall, health professionals considered their new goal setting method to be more patient-centered and meaningful than their earlier practices. Often the patient’s main goal was to return home and recover health. In long-term wards in nursing homes, goals more often related to well-being than recovery. Patients’ relatives were not included in goal setting as a routine, but relatives often expressed their opinions about the goals. The realization of an ideal model of goal setting, could be hampered by shortcomings of both the health services’ ability to tailor services to each patient, and older patients’ capacities to collaborate in the goal setting process. Health professionals’ practices for goal setting with older patients with multi-morbidity comprised four approaches:

### Motivating for goals

Health professionals discussed that some patients could not immediately articulate goals and needed introduction to the goal-setting mindset before they could collaborate with health professionals in goal setting. They educated these patients to take an active role in order to meet health services’ expectations regarding the setting of goals. Moreover, they provided information to patients about services that might help them attain their goals or remain independent. Some patients were passive in goal setting. Health professionals found that older patients were not used to being asked about their preferences regarding health care, wanted to leave decisions to health professionals, or found goal setting difficult.

“They aren’t used to thinking along those lines. Like when we brought in this questionnaire, some people kind of shut down. They didn’t know how to answer, didn’t know, ‘Oh, heavens, I don’t know about that, no, you have to answer that one’ *(laughs)”.* (Nurse at nursing home, Group 3)

Some patients appeared to have the mindset that they had reached a turning point in their age, so that setting rehabilitation goals no longer felt appropriate or like it should be their responsibility. Moreover, some had limited understanding of the current health system.

“Those over 80 are familiar with the old healthcare system, where you stayed at the hospital and you got well. There’s a lot of confusion surrounding the current system. After one or two nights as an in-patient they get discharged to the municipal health services.” (Physical therapist at rehabilitation ward, Group 4)

Health professionals discussed expectations regarding the patient’s role of being active in the rehabilitation process, as the services demand patient effort. They explained to patients the scope for goals and that there would be municipal health services available to assist them towards their goals. Simultaneously, they asked, ‘What matters to you?’ and expected patients to collaborate.

“With those who aren’t motivated—those who aren’t used to thinking that way—with them it’s very important that you try to be a part of their journey and say, ‘What is important to you?’ To try to make clear ‘How will you reach that goal, or what’s important? How will you achieve that?’.” (Physical therapist at rehabilitation ward, Group 4)

Patients were encouraged to set goals such as managing to live in their own homes instead of in an institution. Some patients were tired and less motivated after prolonged illness. Some were reluctant to go home and needed support to focus on their own resources:

“It’s important to focus on what the patients can do on their own, because they’re very—especially after a hospital stay and if they are over 80, then we see it even more—they are very apprehensive, have very little confidence about coming home and have a lot of thoughts about it, and have imagined different scenarios in their heads.” (Physical therapist in reablement, Group 1).

If explaining expectations to patients did not encourage them to collaborate, health professionals set a rehabilitation goal that was earlier than what the patients felt ready for because they had to keep within the timeframe of the service the patient had been allocated. Furthermore, they refrained from performing tasks for patients that they knew the patients were able to perform them themselves in order to help patients understand that they were responsible for doing their part. This felt like a dilemma. To encourage reluctant patients to set goals, health professionals needed a uniform culture among staff regarding patient self-management. When some of the staff did not acknowledge the benefits of support for self-management, they did not expect patients to have such goals. Through giving patients time to consider and expressing empathy for their situations, health professionals found that when patients grasped the mindset, they became motivated to collaborate in goal setting and to perceive the goals as their own.

### Vicariously setting goals

For some patients, health professionals set goals vicariously. At the point when goal setting should have taken place, some older patients with complex needs neither articulated any goals nor made explicit their need for help. These patients’ preferences and needs, or the key factors that could improve their situations, remained unknown. Some patients were ‘in their own foggy world’ because of disease symptoms, such as apathy related to exhaustion, depression, or cognitive impairment. Such symptoms led to challenges for communication. Other patients were in a process of adapting to their disease and so were not ready to set goals or receive help. Some patients covered up their need for help to maintain their social status as independent. Two participants in focus group 2 discussed these issues:

“P6: Particularly people who are so old they don’t want to be a burden on anyone, they want to manage on their own and might conceal their needs.

P1: Either that or they were highly functional people before they became ill. We have a patient like that, a woman with advanced Alzheimer’s who is currently receiving no services. We have tried to go in there but are met with a closed door, ‘No, I don’t want anything’.” (P6, worker without health education, and P1, head nurse, home care services)

Health professionals could not carry out the goal setting procedure in the standardized way with patients whose disease symptoms dictated that they could not take responsibility for setting goals. To elicit what mattered to patients, health professionals identified patients’ problems by establishing a trusting relationship. This meant they were present, observed patients and their surroundings, and got to know them. They also collaborated with patients’ relatives to obtain more information. Subsequently, they set goals vicariously for these patients that they judged might be reasonable for their conditions, for example for a patient with dementia.

“P1: So when she went home with GPS [Global Positioning System] soles in her shoes, it was with her family’s blessing (...)

P2: I think that was important to her.

P1: Yes, it is. And she loves that freedom.” (P1, head nurse, and P2, nurse, home care services, group 1).

In other cases, health professionals deferred goal setting until the symptoms that hampered communication diminished. However, health professionals’ attitudes towards the possibility of involving patients with cognitive impairment varied, as did the amount of time they made available. Thus, their efforts varied, and they excluded some patients by not seeking to elicit their goals. For these patients, applying the goal setting procedure in the health services did not change the levels of collaboration compared to past practices.

### Negotiating goals

Sometimes agreement on goals was challenging to obtain, because health professionals and patients had differing expectations about what the goal should be and who should be responsible for its attainment. Patients, and frequently relatives, expected more services than health professionals considered to be usual or necessary in such a case. Patients’ adult children, in particular, frequently interrupted the goal setting by asking for additional services for their kin. They typically felt it would be safer for the patient to stay in an institution, while the patient wanted to live at home. Unrealistic expectations could also occur when patients expressed goals for improving their health that health professionals judged to be physically unachievable or inappropriate to the timeframe.

“Interviewer: Do they need some help identifying the type of goal they can have?

P2: They might. For instance, some say it’s important for them to get up and walk, and if we see that that is unlikely, we still can’t take the hope away from them. Because then they might not want to be with us, or they lose all motivation. But we try to focus on something that would be achievable during the time they are with us, like being able to walk with a mobility aid or whatever else might be important to them.

P3: Same with us, we have a fairly short time frame in that we have four intensive weeks, so it’s a bit limited what can actually be achieved.” (P2, occupational therapist in short-term ward, and P3, physical therapist in reablement, Group 1)

The approach taken by health professionals to negotiations was, firstly, to consider the extent to which adjusting the goal downward (i.e. towards the patient receiving fewer services) by clarifying expectations would reduce the patient’s will to collaborate. To tell patients that their health goals were too ambitious felt like a dilemma and uncomfortable because this could shatter their hope. Next, health professionals initiated a dialogue to negotiate with patients and, if appropriate, relatives. The approach to this dialogue varied from mentioning which services were available to an explicit negotiation dialogue, which felt like conducting a reality orientation about how the health system worked. Clarifying early in the care pathway the services available could prevent such confrontations. Negotiations felt justified, because resources were allocated to benefit all patients. Furthermore, it was considered legitimate to exclude relatives’ preferences, since the goal setting procedure was designed to weight patients’ autonomy over relatives’ opinions. Challenging negotiations with patients’ children sometimes remained unresolved. With patients, on the other hand, health professionals usually converged on a mutually acceptable goal.

“P6: It was suggested we at least meet half-way (...)

Interviewer: And did everything work out for that person?

P6: I don’t know yet. Guess we’re not quite there yet.

P1: I suppose it’s about finding the second-best solution, something we can all live with.” (P6, worker without health education, and P1, head nurse at home care services, Group 2)

### Specifying goals

Health professionals agreed with some patients on their main goals and assisted them in specifying them. In cases of less complexity, the goal and how it should be specified was often easy to define. In other cases, health professionals adopted the approached to goal setting mentioned previously, which led to a goal being specified. Patients’ goals were often to recover or maintain functional abilities and independence. Such goals were in line with the municipal health services’ objectives and made collaboration easier. However, health professionals perceived patients’ goals as diffuse when they contained no specific actions for attainment.

“It is not very specific goals, I think. It’s either getting better or coming home.” (Physician at short-term ward, Group 4).

A goal of going home could be specified through sub-goals like physical exercises and necessary aids for patients to be safe at home. Health professionals set the sub-goals to plan how the team and the patient could work towards the patient’s goal. They perceived the process as collaborative and on the patients’ terms. Premises.

“It’s just helping them to see there are some steps on the journey.” (Physical therapist at rehabilitation ward, Group 4).

To specify patients’ goals, some used goal-setting instruments to help patients reflect on important areas of life within which they could set self-management sub-goals. Health professionals specified these goals both to be motivating for patients and to match the municipal context within which the team worked. When a patient had several diseases, which led to several or conflicting goals, health professionals set aside their own opinions about what to do and clinical guidelines and collaborated instead on what mattered to the patient. To direct resources in response to patients’ preferences could simultaneously facilitate health professionals’ rationing of care.

“It’s so important that the resources are spent on what the user thinks is important. We might have a user with kidney failure and we think, ‘Well, we need to start dialysis then, that’s clear,’ and so on. But for them, that might not be important at all. They want to stay at home as long as possible and have peace and quiet, not travel to the hospital three times a week. Being free of pain, help them feel safe and confident and such, and their focus might be something completely different from what we were thinking.” (Head nurse at home care services, Group 3).

For patients who were discharged early from hospital, goal attainment was unpredictable due to their unstable health and risk of getting worse. In such cases, health professionals involved patients’ relatives in supporting them to feel safe at home or to do their rehabilitation exercises in order to attain the goals. Patients with serious diseases could not set goals of maintaining health. For these patients, goals were set within the domains of well-being and values, according to the practices of advanced care planning and palliative care.

### Sharing responsibility for goal setting

**Fig. 1:**
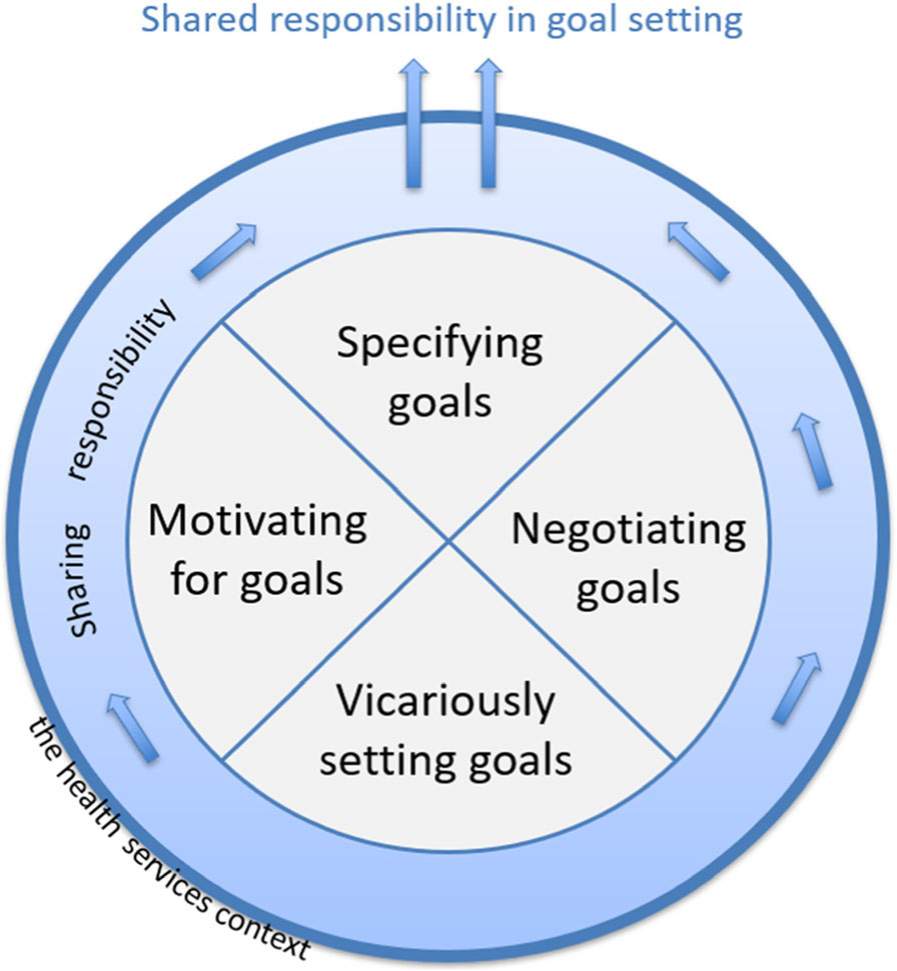
Process of sharing responsibility for goal setting

The core category which contributes to understanding why different approaches were taken to goal setting is health professionals’ sharing of responsibility between patients and the health services. Shared responsibility means that the parties collaborate to agree on goals and contribute within their capacities to attain them. In working with patients whom they perceived as unable to take responsibility for goal setting, health professionals took the ‘vicariously setting goals’ approach (Fig. 1, bottom). The approaches of ‘motivating for goals’ and ‘negotiating goals’ were taken to transfer responsibility for goal setting to patients. This could enable patients to collaborate in the process of specifying goals. ‘Specifying goals’ (at the top of the figure) was the approach taken with the patients perceived as most active, with whom responsibility was most easily shared. The arrows in the figure illustrate the process of sharing responsibility, in which health professionals elicited a commitment from patients to use their own capacities to maintain their health and simultaneously negotiated regarding the contribution the municipal services could make to goal attainment.

These approaches involved an interplay between patients, health professionals, and the health services context. The attitudes of health professionals and the criteria for goal setting varied in wards and contexts. The process was dynamic, and several conversations could occur before a goal was formally defined. Health professionals could draw upon several of the four approaches simultaneously, use the approaches to varying extents, and change approaches. The desired outcome was shared responsibility and agreement on goals.

## Discussion

Collaborative goal setting is a new intervention within integrated care for patients aged 80 and above with multi-morbidity. Health professionals play a vital role in determining how it is implemented and carried out in the health care services. By developing a conceptual model for goal setting approaches, this study adds to existing evidence presenting the four approaches health professionals took to goal setting: motivating for goals, vicariously setting goals, negotiating goals, and specifying goals (Fig. 1). Through these approaches, health professionals shared the responsibility for goal setting with patients. These approaches occurred in an interplay between characteristics of patients, health professionals’ attitudes, and the health service context for the goal setting.

Older patients with multi-morbidity have specific characteristics that must be considered in the process of goal setting [2, 20, 23]. In this study, health professionals perceived patient engagement to be a starting point for goal setting. Patient engagement varied, due to age-related functional decline, unpredictable disease symptoms, and because the older generation lacked knowledge of the health system. Previous studies indicate that health professionals perceive that some patients with multi-morbidity do not naturally articulate goals [8, 23, 26] and that it takes effort to engage them [8]. Health professionals motivate and negotiate goals with patients with single diseases [23–25]. For patients with cognitive impairment, health professionals have previously been found to set goals vicariously [37]. In the present study, health professionals were found to use all these practices, motivating, negotiating, and setting goals vicariously for elderly patients with multi-morbidity. Few studies have reported high levels of patient participation in goal setting [8, 38]. Health professionals’ perceptions that these patients do not wish to be involved are contrary to research showing that older people with multi-morbidity in community settings prefer to participate actively, although, admittedly, to a lesser extent when they have four or more conditions [39]. In our study, health professionals found it easier to set goals with patients with less complex needs; for these patients they used the approach of ‘specifying goals’. Patients’ readiness to be involved, their motivation, and the extent to which they take responsibility, are prerequisites for co-creating care that matters to patients [1, 16, 17].

Facilitating co-production of care that aligns with patients’ formulated goals is emphasized as a strategy in current policy on integrated care [1]. Health professionals in the present study and in other studies perceived their practices as being more oriented towards patients’ preferences after they had begun to set goals with them [8, 35]. However, health professionals argued they needed to be in the driving seat of goal setting. This is in line with studies showing that health professionals tend to align goal setting with perceived responsibilities towards the system or medical knowledge [8, 28]. A new, related finding is that the challenges they experienced in motivating patients to adopt their goals, were related to differing perceptions as to whether patients were responsible for setting goals. Health professionals and older patients had conflicting perceptions of whether patients had reached a turning point in old age after which rehabilitation goals were no longer appropriate to set. Patient participation is often less sought by patients in the acute phase of illness and by patients who have several conditions [8, 39], a finding which this study confirms.

The objectives of integrated care include maintaining older patients’ health, increasing the quality of their care experience through goal setting, and reducing care utilization by having health professionals support patients to live in the community [1, 17]. The health professionals studied here worked towards these objectives, which could, in practice, conflict. They held the attitude that patients’ goals were not always realistic given the limitations in the health service system. Hence, the goals were partly pre-defined by health professionals, to suit the limited timeframe of municipal health services and the objective of maintaining patients’ health in order to allow them to manage at home. Thus, there is a risk that responsibility for the attainment of the health services’ goals for independence could be transferred to individual patients [40], possibly against the will or capacity of older patients with multi-morbidity. When health professionals and patients disagree on the desired outcomes of service delivery, co-destruction rather than co-creation of value is likely to happen [15, 19].

### Implications for policy and health services

The current intervention for collaborative goal setting is introduced to enhance patient participation, service outcome and satisfaction with service delivery. However, the potential conflicts which can occur in such goal setting, should be considered in future health policies. Current health reforms aim to move care for complex patients out of hospitals [17], increasingly aiming for ageing in place and care and treatment in the municipal context. Following, conflicts in in goal setting could increase in the future. Also, by the increased focus on activity and reablement, goal setting instruments could increasingly transfer responsibility for outcomes of service delivery to patients. This will be an unintended consequence of the health political objectives of co-production of service delivery. As shown in this study, health care professionals spend a lot of time on the collaborative goal setting intervention, both conducting the structured conversation with patients, as well as documenting and following up the goals. To conclude whether this goal setting is worth spending professional time on, more studies are needed. Both quantitative studies examining the level of patient participation in goal setting models should be performed, as well as studies focusing on the potential changes in service delivery, service outcome and patient satisfaction following the new practice. In times when health care professionals are becoming a limited resource, implementation of time-consuming interventions should be followed by evaluation of their effects. In Norway, as in other countries, effect studies of interventions in municipal health services are limited and sought for [41].

This study has two implications for health services. Firstly, the conceptual model of approaches to goal setting created here, could be used in education and clinical settings, for health professionals to increase reflections and consciousness about how to tailor goal setting to the diverse group of patients with multi-morbidity, and on the extent to which patients should be given responsibility for determining their goals and the care services they need. Secondly, at the health service system level, our findings indicate that even though clinical guidelines to increase participation for patients with multi-morbidity is developed [1–3, 11], the goal setting tools used in clinical practice could be further developed to specify different approaches, that account for patients’ level of disease severity and ability for participation. Further research could refine our model of four approaches to goal setting in other health service settings. Moreover, other possible mechanisms than sharing responsibility, which also may influence the goal setting, could be explored.

### Limitations of the study

Few situations in which patients were excluded from goal setting were described. This could be since the participants were interviewed in a group with colleagues, and six participants were in groups with their managers. In two of the groups, participants had been asked by their managers to participate, and we do not know whether the most positive workers were chosen. These factors could have led participants to describe their efforts to involve patients in a more positive way. Furthermore, health professionals demonstrated a strong focus on setting goals for independence. Three-quarters of the participants worked within rehabilitation services. Therefore, the results may be less transferable to long-term services in nursing homes, since goals in those contexts can cover other dimensions, such as well-being [20]. We do, however, suggest that the results provide a general perspective for understanding goal setting, for the increasing and fragile group of older patients with multi-morbidity, both across countries and different care settings.

## Conclusions

In collaborative goal setting with patients aged 80 and above with multi-morbidity, municipal health professionals to a varying extent shared responsibility for service delivery with each patient. To agree on goals, health professionals took four approaches: motivating for goals, vicariously setting goals, negotiating goals, and specifying goals. Goals were co-produced in an interplay of patient characteristics that influenced their engagement and health professionals’ attitudes regarding who should be responsible for goal setting. Health professionals’ processes of sharing responsibility with patients reflect the ambiguous objectives of both improving patients’ perceptions of quality of care and reducing care utilization, which is found in health policy and municipal health services. These ambiguous objectives for goal setting could lead to reduced collaboration on what matters to patients and ultimately circumscribe the role of the patient in co-producing service delivery.

## Supplementary information

**Additional file 1.** Interview guide.

## Data Availability

The data generated and analyzed in the current study are not publicly available due to Norwegian privacy legislation and the form signed by the participants about the study’s privacy. The data generated are available from the corresponding author on reasonable request.

## References

[1] World Health Organization (2016). Framework on integrated, people-centred health services: report by the secretariat.

[2] Leijten FRM, Struckmann V, van Ginneken E, Czypionka T, Kraus M, Reiss M (2018). The SELFIE framework for integrated care for multi-morbidity: development and description. Health Policy..

[3] Palmer K, Marengoni A, Forjaz MJ, Jureviciene E, Laatikainen T, Mammarella F (2018). Multimorbidity care model: recommendations from the consensus meeting of the joint action on chronic diseases and promoting healthy ageing across the life cycle (JA-CHRODIS). Health Policy..

[4] Smith SM, Wallace E, O'Dowd T, Fortin M (2016). Interventions for improving outcomes in patients with multimorbidity in primary care and community settings. Cochrane Libr.

[5] Xu X, Mishra GD, Jones M (2017). Evidence on multimorbidity from definition to intervention: an overview of systematic reviews. Ageing Res Rev.

[6] de Bruin SR, Versnel N, Lemmens LC, Molema CCM, Schellevis FG, Nijpels G (2012). Comprehensive care programs for patients with multiple chronic conditions: a systematic literature review. Health Policy.

[7] OECD 2011. Future demographic trends and long-term care costs. In: Help wanted? Providing and paying for long-term care. https://www.oecd.org/els/health-systems/47884543.pdf accessed 20 Nov 2019.

[8] Rose A, Rosewilliam S, Soundy A (2017). Shared decision making within goal setting in rehabilitation settings: a systematic review. Patient Educ Couns.

[9] Berntsen GKR, Gammon D, Steinsbekk A, Salamonsen A, Foss N, Ruland C (2015). How do we deal with multiple goals for care within an individual patient trajectory? A document content analysis of health service research papers on goals for care. BMJ Open.

[10] Kuluski K, Gill A, Naganathan G, Upshur R, Jaakkimainen RL, Wodchis WP (2013). A qualitative descriptive study on the alignment of care goals between older persons with multi-morbidities, their family physicians and informal caregivers. BMC Fam Pract.

[11] National Institute for Health and Care Excellence. Multimorbidity: Clinical assessment and management. NICE guideline (NG56). United Kingdom; 2016. https://www.nice.org.uk/guidance/ng56. Accessed 20 Nov 2019.

[12] The Norwegian Directorate of Health. Veileder om oppfølging av personer med store og sammensatte behov (Follow-up of people with large and complex needs) 2018 [cited 2018 02.22]. https://helsedirektoratet.no/retningslinjer/oppfolging-av-personer-med-store-og-sammensatte-behov. Accessed 20 Nov 2019.

[13] Lenzen Stephanie Anna, Daniëls Ramon, van Bokhoven Marloes Amantia, van der Weijden Trudy, Beurskens Anna (2017). Disentangling self-management goal setting and action planning: A scoping review. PLOS ONE.

[14] Berntsen GKR, Dalbakk M, Hurley JS, Bergmo T, Solbakken B, Spansvoll L, Bellika JG, Skrøvseth SO, Brattlend T and Rumpsfeld M. Person-centred, integrated and pro-active care for multi-morbid elderly with advanced care needs: a propensity score-matched controlled trial. BMC Health Serv Res. 2019 19:682. doi.org/10.1186/s12913-019-4397-2 10.1186/s12913-019-4397-2 PMC6777026 31581947

[15] Palumbo R (2016). Contextualizing co-production of health care: a systematic literature review. Public Sect Manag.

[16] Osborne Stephen P, Radnor Zoe, Strokosch Kirsty (2016). Co-Production and the Co-Creation of Value in Public Services: A suitable case for treatment?. Public Management Review.

[17] WHO. Innovative Care for Chronic Conditions: Building Blocks for Action. Geneva: World Health Organisation (WHO); 2002. https://www.who.int/chp/knowledge/publications/icccglobalreport.pdf. Accessed 20 Nov 2019

[18] WHO (2015). People-centred and integrated health services: an overview of the evidence Interim Report. Secondary People-centred and integrated health services: an overview of the evidence. Interim report.

[19] Palumbo R, Manna R (2017). What if things go wrong in co-producing health services? Exploring the implementation problems of health care co-production. Policy and Soc.

[20] Vermunt NPCA, Harmsen M, Westert GP, Olde Rikkert MGM, Faber MJ (2017). Collaborative goal setting with elderly patients with chronic disease or multimorbidity: a systematic review. BMC Geriatr.

[21] Berntsen GR, Høyem A, Lettrem I, Ruland C, Rumpsfeld M, Gammon DB (2018). A person-centered integrated care quality framework, based on a qualitative study of patients’ evaluation of care in light of chronic care ideals. BMC Health Serv Res.

[22] Lenzen SA, Daniëls R, van Bokhoven MA, van der Weijden T, Beurskens A. Development of a conversation approach for practice nurses aimed at making shared decisions on goals and action plans with primary care patients. BMC Health Serv Res; London. 2018;Vol 18. 10.1186/s12913-018-3734-1. 10.1186/s12913-018-3734-1 PMC6258162 30477566

[23] Boeckxstaens P, Willems S, Lanssens M, Decuypere C, Brusselle G, Kühlein T (2016). A qualitative interpretation of challenges associated with helping patients with multiple chronic diseases identify their goals. J Comorbidity.

[24] Bodenheimer T, Handley MA (2009). Goal-setting for behavior change in primary care: an exploration and status report. Patient Educ Couns.

[25] Scobbie L, Wyke S, Dixon D (2009). Identifying and applying psychological theory to setting and achieving rehabilitation goals. Clin Rehabil.

[26] Franklin M, Lewis S, Willis K, Bourke-Taylor H, Smith L (2017). Patients’ and healthcare professionals’ perceptions of self-management support interactions: systematic review and qualitative synthesis. Chronic Illness.

[27] Lenzen SA, van Dongen JJJ, Daniëls R, van Bokhoven MA, van der Weijden T, Beurskens A (2016). What does it take to set goals for self-management in primary care? A qualitative study. Fam Pract.

[28] Levack WMM, Dean SG, Siegert RJ, McPherson KM (2011). Navigating patient-centered goal setting in inpatient stroke rehabilitation: how clinicians control the process to meet perceived professional responsibilities. Patient Educ Couns.

[29] Angel S, Frederiksen KN (2015). Challenges in achieving patient participation: a review of how patient participation is addressed in empirical studies. Int J Nurs Stud.

[30] Charmaz K (2014). Constructing grounded theory.

[31] Krueger RA, Casey MA (2015). Focus groups: a practical guide for applied research.

[32] Cochrane A, Furlong M, Mcgilloway S, Molloy D, Stevenson M, Donnelly M (2016). 070 the effects of time-limited home-care reablement services for older people: a cochrane systematic review. Age Ageing.

[33] The Norwegian Directorate of Health. Veileder for saksbehandling 2017. (Guideline for case management). https://helsedirektoratet.no/retningslinjer/veileder-for-saksbehandling-av-tjenester-etter-helse-og-omsorgstjenesteloven. Accessed 20 Nov 2019.

[34] Helse-, omsorgs- og rehabiliteringsstatistikk. Eldres helse og bruk av kommunale helse- og omsorgstjenester. Rapport IS-2375. (Health- care and rehabilitation statistics, report) The Norwegian Directorate of Health, 2016. https://www.helsedirektoratet.no/rapporter/helse-omsorgs-og-rehabiliteringsstatistikk-eldres-helse-og-bruk-av-kommunale-helse-og-omsorgstjenester/Helse,-omsorgs,-%20og%20rehabiliteringsstatistikk%20%E2%80%93%20Eldres%20helse%20og%20bruk%20av%20kommunale%20helse-%20og%20omsorgstjenester.pdf/ Accessed 20 Nov 2019.

[35] Ervik R, Lindèn TS, Askildsen JE (2016). S R. SELFIE 2020 Work Package 2: Thick descriptions of Learning networks for whole, coordinated and safe pathways (Learning networks).

[36] Thornberg R, Charmaz K. Grounded theory and theoretical coding. 2014 2019/04/01. In: The SAGE handbook of qualitative data analysis [internet]. London: SAGE Publications Ltd. https://methods.sagepub.com/book/the-sage-handbook-of-qualitative-data-analysis. Accessed 20 Nov 2019.

[37] Dörfler E, Kulnik ST. Despite communication and cognitive impairment – person-centred goal-setting after stroke: a qualitative study. Disabil Rehabil. 2019. 10.1080/09638288.2019.1604821. 10.1080/09638288.2019.1604821 31020863

[38] Sugavanam T, Mead G, Bulley C, Donaghy M, van Wijck F (2013). The effects and experiences of goal setting in stroke rehabilitation—a systematic review. Disabil Rehabil.

[39] Chi WC, Wolff J, Greer R, Dy S (2017). Multimorbidity and decision-making preferences among older adults. Ann Fam Med.

[40] Christensen K, Fluge S (2016). User participation in Norwegian elderly care policy – The development of rhetoric about individual responsibility. (Norwegian title: Brukermedvirkning I norsk eldreomsorgspolitikk – Om utviklingen av retorikken om individuelt medansvar). Tidsskrift Velferdsforskning.

[41] Flottorp S, Harboe I, Kornør H, Sandberg H, Smedslund G (2019). Omsorgstjenesteforskningen i Norge (Care Services Research in Norway). The Research Council of Norway.

