# Supplementary material for: Sharing responsibility: municipal health professionals’ approaches to goal setting with older patients with multi-morbidity – a grounded theory study
Source: BMC Health Serv Res. 2020 Feb 24;20:141. doi: 10.1186/s12913-020-4983-3 (PMC7041090; doi:10.1186/s12913-020-4983-3)
Supplement: Supplementary file 1 — Additional file 1. Interview guide. [file 12913_2020_4983_MOESM1_ESM.docx]

Additional file 1: Interview guide

| **Interview guide** |
| --- |
| Can you describe what you do when you ask patients ‘What matters to you?’  Could you describe situations in which you asked, ‘What matters to you?’ and the patients’ answer had implications for the help you gave?  Could you describe situations in which you asked, ‘What matters to you?’ and the patients’ answer had minor implications for the help you gave?  Do the patients participate differently than before you implemented the ‘What matters to you?’ procedure? |
| **Theoretical sampling questions in the latest interviews** |
| What should one do when patients do not envisage any goals?  Which criteria do you consider when you say that a patient’s goal is realistic?  Do the relatives also express what matters to the patient? |
